# Genetically modified pigs as donors of cells, tissues, and organs for xenotransplantation

**DOI:** 10.1093/af/vfz014

**Published:** 2019-06-25

**Authors:** Eckhard Wolf, Elisabeth Kemter, Nikolai Klymiuk, Bruno Reichart

**Affiliations:** 1 Chair for Molecular Animal Breeding and Biotechnology, Gene Center and Department of Veterinary Science, LMU Munich, Munich, Germany; 2 German Center for Diabetes Research (DZD), Neuherberg, Germany; 3 Walter Brendel Center for Experimental Medicine, LMU Munich, Germany; 4 German Center for Cardiovascular Research (DZHK), Partner Site Munich, Germany

**Keywords:** heart, organ donor, pancreatic islet, pig, xenotransplantation

**Implications**

- For many patients with chronic organ failure, transplantation is the only therapeutic option, but the number of donated human organs and tissues falls far short of the need.
- Porcine cells, tissues, and organs likely will be an alternative transplant source, since pigs can be genetically engineered to overcome rejection mechanisms and physiological incompatibilities, and to reduce the risk of transmitting zoonotic pathogens.
- Significant progress has been made in many areas of xenotransplantation, including pancreatic islets, neuronal cells, and corneas, but also vascularized organs, especially kidneys and hearts.
- In view of recent preclinical breakthroughs, such as consistent long-term survival of baboons after orthotopic transplantation of a genetically multimodified porcine heart, xenotransplantation can be considered as a realistic future therapeutic option.

## Introduction

The number of donated human organs and tissues for patients with terminal organ failure falls far short of the need. According to the United Network for Organ Sharing (www.unos.org), more than 113,000 candidates for transplant are currently on the U.S. national waiting list, but only 36,527 organ transplants could be performed in 2018. Alternative sources, such as organs and tissues from animals, are therefore urgently needed. For a number or reasons, including size, anatomical, and physiological similarities with humans, the pig is the preferred donor species (reviewed in Cooper et al., 2016). Importantly, pigs can be optimized by genetic engineering as a source of cells, tissues, and organs for xenotransplantation. Recent advances in gen(om)e editing are speeding up progress in this field. Numerous genetically (multi-)modified pig lines have been generated to prevent immune rejection of xenotransplants, to overcome physiological incompatibilities, and to reduce the risk of transmitting zoonotic pathogens (Table 1; reviewed in Kemter et al., 2018).

**Table 1.** Selection of genetic modifications of donor pigs for xenotransplantation

Aim/Genetic modification (GM)	Reference
Deletion of sugar moieties of pig cells with pre-formed recipients’ antibodies	
α-1,3-galactosyltransferase knockout (GGTA1-KO)	(Phelps et al., 2003)
Cytidine monophosphate-N-acetylneuraminic acid hydroxylase knockout (CMAH-KO)	(Kwon et al., 2013; Lutz et al., 2013)
β-1,4-N-acetyl-galactosaminyl transferase 2 knockout (B4GALNT2-KO)	(Estrada et al., 2015)
Complement regulation by human complement-regulatory gene expression	
Human membrane cofactor protein transgenic (hCD46-tg)	(Diamond et al., 2001)
Human decay-accelerating factor transgenic (hCD55-tg)	(Cozzi and White, 1995)
Human protectin or membrane inhibitor of reactive lysis transgenic (hCD59-tg)	(Fodor et al., 1994)
Human complement-regulatory protein C1 inhibitor transgenic (hC1-INH-tg)	(Kwon et al., 2017)
Coagulation regulation by human coagulation-regulatory gene expression	
Human thrombomodulin transgenic (hTBM-tg)	(Wuensch et al., 2014)
Human endothelial protein C receptor transgenic (hEPCR-tg)	(Iwase et al., 2014)
Human tissue factor pathway inhibitor transgenic (hTFPI-tg)	(Lin et al., 2010)
Human ectonucleoside triphosphate diphosphohydrolase-1 transgenic (hCD39-tg)	(Wheeler et al., 2012)
Human ecto-5′-nucleotidase transgenic (hCD73-tg)	(Lee et al., 2017)
Prevention of cell-mediated rejection - T cells	
Human LEA29Y transgenic (LEA29Y-tg)	(Klymiuk et al., 2012; Bähr et al., 2016)
Human CTLA4-Ig transgenic (hCTLA4-Ig-tg)	(Martin et al., 2005)
Porcine CTLA4-Ig transgenic (pCTLA4-Ig-tg)	(Phelps et al., 2009)
SLA class I knockout	(Reyes et al., 2014)
Human dominant-negative mutant class II transactivator transgenic (CIITA-DN-tg)	(Hara et al., 2013)
Human TNF-related apoptosis-inducing ligand transgenic (hTRAIL-tg)	(Klose et al., 2005)
Human-programmed cell death 1 ligand 1 transgenic (PD-L1-tg)	(Buermann et al., 2018)
Prevention of cell-mediated rejection - natural killer cells and macrophages	
HLA-E/human b2-microglobulin transgenic (HLA-E/b2M-tg)	(Weiss et al., 2009)
Human CD47 transgenic (hCD47-tg)	(Tena et al., 2014)
Expression of anti-inflammatory proteins or knockout of pro-inflammatory proteins	
Human tumor necrosis factor α–induced protein 3 (TNFAIP3) transgenic (A20-tg)	(Oropeza et al., 2009)
Human heme oxygenase 1 transgenic (hHO-1-tg)	(Petersen et al., 2011)
Soluble human TNFRI-Fc transgenic (shTNFRI-Fc-tg)	(Yan et al., 2016)
Reduction/elimination of the risk of PERV transmission	
Knockdown of PERV expression	(Miyagawa et al., 2005; Dieckhoff et al., 2008; Ramsoondar et al., 2009)
Genome-wide inactivation of PERV pol gene	(Niu et al., 2017)
Genetically multimodified pigs	
GGTA1-KO/hCD46-tg/hCD39-tg	(Bottino et al., 2014)
GGTA1-KO/hCD46-tg/hTFPI-tg/pCTLA4-Ig-tg	(Bottino et al., 2014)
GGTA1-KO/hCD46-tg/hTFPI-tg/pCTL4-Ig-tg/hCD39-tg	(Bottino et al., 2014)
GGTA1-KO/hCD55-tg/hCD59-tg/human fucosyltransferase (HT)-tg	(Le Bas-Bernardet et al., 2011)
GGTA1-KO/hCD55-tg/hCD59-tg	(Hawthorne et al., 2014)
GGTA1-KO/hCD55-tg/hCD39-tg/TFPI-tg/hC1-INH-tg/hTNFAIP3-tg	(Kwon et al., 2017)
GGTA1-KO/CMAH-KO/hCD46-tg/hCD55-tg/hCD59-tg/hA20-tg/hHO1-tg	(Fischer et al., 2016)

### Genetic modifications to overcome hyperacute and acute vascular rejection of pig-to-primate xenotransplants

Hyperacute rejection of vascularized pig-to-primate xenotransplants is triggered by binding of preformed antibodies of the recipient to specific antigens on the xenogeneic tissue and subsequent activation of the complement system. The major xeno-antigen is galactose-α1,3-galactose (αGal) synthesized by α-1,3-galactosyltransferase (GGTA1). Humans and Old World monkeys lack GGTA1 and αGal epitopes, but are exposed to bacterial αGal epitopes eliciting a persistent anti-αGal antibody response in early life. Other prominent xeno-antigens are N-acetylneuraminic acid (Neu5Gc) synthesized by cytidine monophosphate-N-acetylneuraminic acid hydroxylase (CMAH) and an Sd(a)-like glycan made by porcine β-1,4-N-acetyl-galactosaminyl transferase 2 (B4GALNT2) (reviewed in Byrne et al., 2015).

An important step toward long-term survival of vascularized porcine xenotransplants in nonhuman primates was the generation of pigs lacking functional *GGTA1* alleles (Phelps et al., 2003). Subsequently, multiple *GGTA1* knockout pig lines were generated, initially by gene targeting (reviewed in Klymiuk et al., 2010) and later by gene editing (e.g., Hauschild et al., 2011). In addition, pigs with knockout mutations of *CMAH*, *B4GALNT2* or combinations of these modifications were generated (Estrada et al., 2015). The authors showed that cells from GGTA1/CMAH/B4GALNT2–deficient pigs exhibited reduced human IgM and IgG binding compared with cells lacking only GGTA1 and CMAH.

A complementary strategy is the generation of transgenic pigs that express human complement-regulatory proteins, such as CD46 (membrane cofactor protein, MCP), CD55 (complement decay-accelerating factor, DAF), or CD59 (membrane inhibitor of reactive lysis, MIRL), singly or in combination. These complement-regulatory proteins attenuate complement activation and significantly prolong survival of pig-to-nonhuman primate xenotransplants (reviewed in Cooper et al., 2016).

By combination of *GGTA1* knock-out with the expression of one or more human complement-regulatory proteins, the problem of hyperacute rejection of porcine xenotransplants in nonhuman primates has been solved. For clinical trials, additional knock-outs of *CMAH* and *B4GALNT2* may be required (reviewed in Kemter et al., 2018).

Besides preformed antibody binding to carbohydrate antigens, xenotransplantation of porcine cells, tissues, or organs elicits a humoral immune response (reviewed in Vadori and Cozzi, 2015). The risk is likely increased in presensitized patients with antibodies against major histocompatibility complex (MHC) class I molecules/human leukocyte antigens, since these antibodies may cross-react with conserved epitopes of swine MHC subclasses/swine leukocyte antigens (Mulder et al., 2010). To overcome this problem, pigs lacking MHC class I have been generated. These pigs showed reduced levels of CD4^−^ CD8^+^ T cells in the peripheral blood, but appeared healthy and developed normally (Reyes et al., 2014).

### Genetic modifications to overcome cellular rejection of pig-to-primate xenotransplants

Cellular rejection of pig-to-primate xenotransplants involves both innate and adaptive components of the cellular immune system. Immune cell infiltration of tissue and solid organ xenotransplants starts with neutrophils, followed by macrophages and T cells (reviewed in Vadori and Cozzi, 2015). In addition, natural killer cells may induce endothelial cell activation in the xenotransplant and lyse porcine cells directly and via antibody-dependent cytotoxicity (reviewed in Weiss et al., 2009).

Cellular xenotransplants such as porcine islets in nonhuman primates are mainly rejected by CD4^+^ T cells. Their activation can be induced by direct binding of primate T-cell receptors to swine leukocyte antigen class 1 and class 2 molecules of porcine cells, or indirectly by antigen-presenting cells of the recipient expressing MHCs with processed xeno-antigens (reviewed in Vadori and Cozzi, 2015). In addition, co-stimulatory signals, which may induce and amplify an effective immune response, or exhibit an inhibitory function, are involved in the regulation of T-cell function. The most prominent T-cell co-stimulatory signaling complexes are CD40 (on APCs)-CD154 (on T cells) and CD80/CD86 (on antigen-presenting cells)-CD28 (on T cells). The CD80/CD86-CD28 co-stimulation pathway can be blocked by systemic treatment with CTLA4-Ig (abatacept) or its affinity-optimized version LEA29Y (belatacept) (reviewed in Bartlett et al., 2016). These molecules can also be expressed in genetically modified donor pigs, opening the prospect of inhibiting T-cell activation locally at the graft site, thus avoiding systemic immunosuppression of the recipient and the consequent risk of infection. Protective effects of human CTLA4-Ig expression on porcine cells and tissues were shown in xenogeneic neuronal cell (Aron Badin et al., 2016) and skin transplantation experiments (Wang et al., 2015).

**Figure 1.**
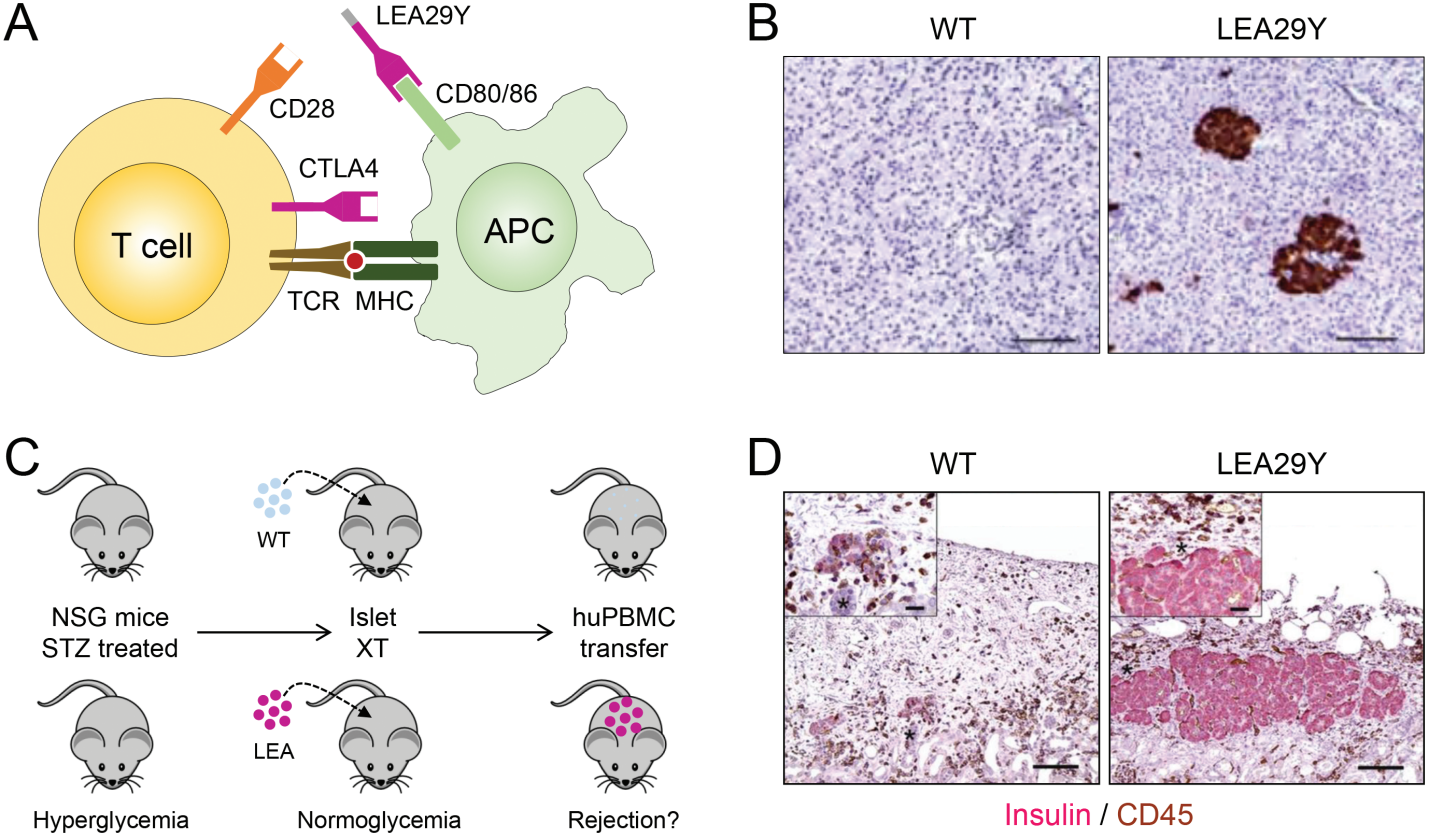
Protection of xenotransplanted porcine pancreatic islets against T-cell mediated rejection by local expression of LEA29Y. (A) Principle of co-stimulation blockade of T cells. Activation of T cells requires interaction between the T-cell receptor and a peptide-loaded MHC on an antigen-presenting cell (APC). In addition, a second signal such as the interaction between CD28 und CD80/CD86 is required. The interaction of CTLA4 and CD80/CD86 blocks T-cell activation. The latter can also be achieved by the soluble molecule CTLA4-Ig or its affinity-optimized version LEA29Y. (B) Immunohistochemical staining of LEA29Y in pancreas sections from INS-LEA29Y transgenic pigs. (C) Transplantation of neonatal islet-like cell clusters (NICCs) from wild-type (WT) or INS-LEA29Y transgenic pigs (LEA29Y) under the kidney capsule of immune deficient streptozotocin (STZ)-diabetic NSG mice results in an insulin-positive cell mass that is able to normalize their blood glucose level. If the mice are subsequently reconstituted with human peripheral blood mononuclear cells (hPBMCs), the WT islets are rejected while the LEA29Y transgenic islets are protected (Klymiuk et al., 2012). (D) Histology of the transplantation site. In recipients of WT NICCs, very few insulin-positive cells were found, but a massive T-cell infiltration (shown by CD45 staining) was evident. In contrast, LEA29Y expressing NICCs survived and formed large clusters of insulin-positive cells. T-cell infiltration was observed in the periphery, but not within the insulin-positive cell clusters.

LEA29Y expressing transgenic porcine neonatal islet-like cell clusters transplanted into immunodeficient diabetic mice normalized blood glucose levels and, in contrast to wild-type neonatal islet-like cell clusters, were not rejected after the recipient mice were reconstituted with human immune cells (Figure 1) (Klymiuk et al., 2012). A subsequent study using diabetic mice with a long-term “humanized” immune system as recipients showed that LEA29Y expressing porcine neonatal islet-like cell clusters survived for several months and normalized the recipients’ blood glucose levels, whereas wild-type islets did not engraft in this model (Wolf-van Buerck et al., 2017). Neonatal islet-like cell clusters have a number of advantages over adult porcine islets, most importantly their straightforward isolation, their proliferation capacity, their superior revascularization after transplantation, and the fact that donor animals do not need to be maintained for a long period under expensive designated pathogen-free conditions (reviewed in Kemter and Wolf, 2018). However, neonatal islet-like cell clusters are immature and not fully functional after isolation. To visualize the maturation and proliferation of neonatal islet-like cell clusters, we generated transgenic pigs expressing enhanced green fluorescent protein (eGFP) under the control of the porcine insulin gene (*INS*) promoter. The reporter gene is expressed specifically in beta cells and the level of expression increases upon beta-cell maturation (Kemter et al., 2017). This model is useful to study beta-cell maturation and expansion in vivo, e.g., after transplantation into the anterior eye chamber of mice. Moreover, eGFP-expressing beta cells can be recovered by fluorescence-activated cell sorting and processed for molecular profiling studies, such as single-cell RNA sequencing (Kemter and Wolf, 2018).

To prevent lysis of xenogeneic cells by natural killer cells, transgenic pigs expressing HLA-E/beta2-microglobulin were generated. Their cells were effectively protected against human natural killer-cell mediated cytotoxicity, depending on the level of CD94/NKG2A expression on the natural killer cells (Weiss et al., 2009). To control macrophage activity, human CD47 has been expressed on porcine cells to activate the “don’t eat me signal” receptor SIRPα on (human) monocytes/macrophages and to suppress phagocytic activity (reviewed in Cooper et al., 2016).

### Genetic modifications to overcome dysregulation of coagulation and inflammation

Dysregulation of coagulation and disordered hemostasis are frequent complications in preclinical pig-to-nonhuman primate xenotransplantation studies. Inflammation, vascular injury, innate, humoral and cellular immune responses, and, in particular, molecular incompatibilities between porcine and primate regulators of coagulation are discussed as potential causes (reviewed in Cowan and Robson, 2015).

**Figure 2.**
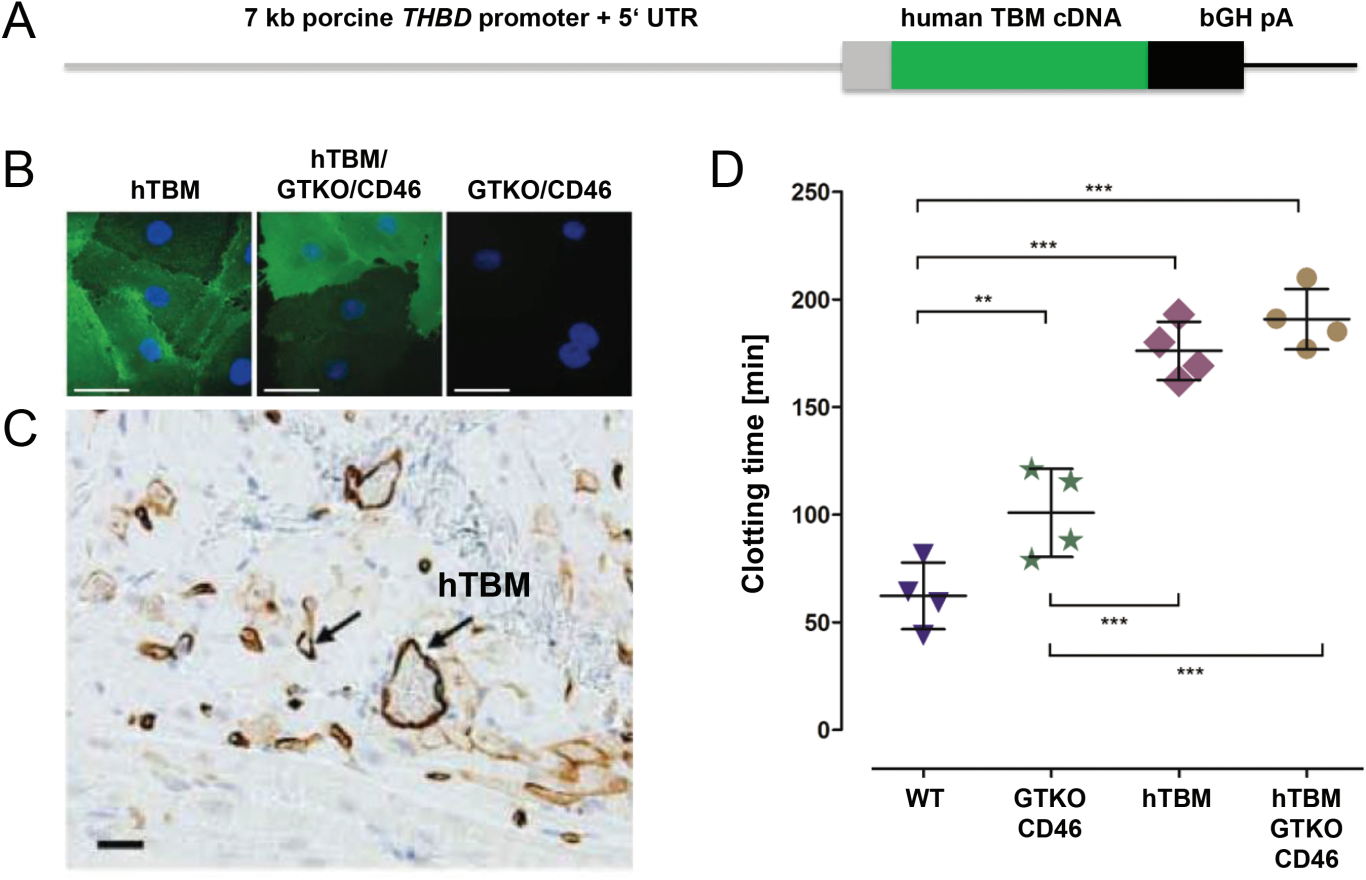
Expression of human thrombomodulin (hTBM) in genetically (multi-)modified pigs. (A) Expression vector with the porcine *THBD* gene promoter. (B) Immunofluorescence staining of hTBM in transgenic porcine endothelial cells. (C) Expression of hTBM in vascular endothelial cells of myocardium from transgenic pigs. (D) Beads covered with hTBM expressing endothelial cells from genetically (multi-)modified pigs delay clotting of human blood (Wuensch et al., 2014).

Key endothelial anticoagulant/antithrombotic proteins that have been modified/supplemented by genetic engineering of donor pigs include human thrombomodulin (TBM), endothelial protein C receptor, tissue factor pathway inhibitor, and ectonucleoside triphosphate diphosphohydrolase 1 (CD39) (reviewed in Cowan and Robson, 2015). Porcine thrombomodulin binds human thrombin, but is a poor co-factor for activation of human protein C. Therefore, we generated transgenic pigs expressing human thrombomodulin under the control of the porcine thrombomodulin gene (*THBD*) promoter (Figure 2) (Wuensch et al., 2014). A *GGTA1* knockout, hCD46/hTBM transgenic pig heart survived for 945 d after heterotopic abdominal transplantation into a baboon with appropriate immunosuppression (Mohiuddin et al., 2016).

In addition to modifications targeting coagulation disorders in xenotransplantation, transgenic pigs expressing antiapoptotic and antiinflammatory proteins, such as human tumor necrosis factor-alpha-induced protein 3 (A20) (Oropeza et al., 2009) and human heme oxygenase-1 (HO-1) (Petersen et al., 2011), have been produced.

### Genetic modifications to decrease the risk for zoonoses

Xenotransplantation may be associated with the risk of transmission of porcine microorganisms including bacteria, fungi, and viruses able to adapt in the recipient and to induce a disease (zoonosis or xenosis) (reviewed in Fishman, 2018). Many microorganisms can be eliminated from the donor pigs by selection, treatment with antibiotics, antimycotics or antiviral drugs, by vaccination, by early weaning and colostrum deprivation, by caesarean delivery or embryo transfer, and by maintenance of the donor animals in designated pathogen-free housing facilities (reviewed in Kemter et al., 2018). An example is the elimination of porcine cytomegalovirus by early weaning of piglets, even if their mothers were infected (Egerer et al., 2018).

In contrast, porcine endogenous retroviruses (PERVs) cannot be eliminated this way, because they are integrated in the genome of all pigs and can be released from pig tissues as infectious virus particles. Until now, no transmission of PERV has been observed in preclinical and clinical trials (Denner, 2018). To prevent PERV transmission despite their integration in the pig genome, several strategies have been developed: 1) selection of pigs with a low copy number and a low expression of PERV-A and PERV-B proviruses; 2) selection of PERV-C free animals to avoid PERV-A/C recombinants with increased replication competence; 3) knockdown of PERV expression by RNA interference in transgenic pigs; and 4) vaccination against transmembrane and surface envelope proteins of PERV (reviewed in Kemter et al., 2018).

A breakthrough was achieved when the CRISPR/Cas9 technology was used to inactivate PERVs integrated in the pig genome. After proof of principle in immortalized PK-15 pig cells (Yang et al., 2015), all PERV copies (altogether 25) were inactivated in primary pig cells and these were used for somatic cell nuclear transfer to produce live healthy piglets (Niu et al., 2017). The technical feasibility of reducing the risk of PERV transmission to zero is exciting, but it is not clear at this stage if genome-wide PERV inactivation by CRISPR/Cas9 is actually required for entering clinical xenotransplantation trials.

### Recent breakthrough in orthotopic pig-to-baboon cardiac xenotransplantation

**Figure 3.**
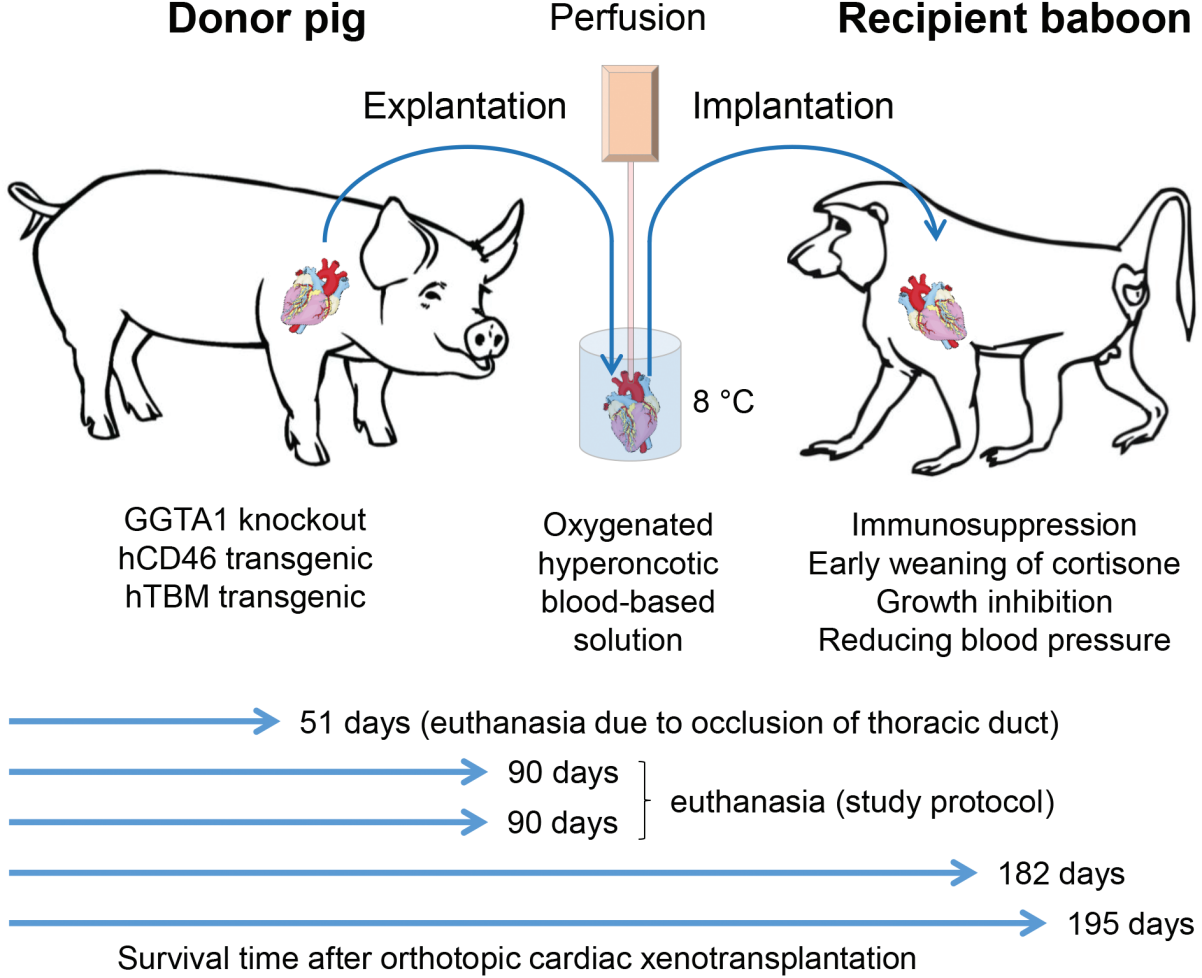
Factors enabling consistent success in life-supporting pig-to-baboon cardiac xenotransplantation. In addition to genetically multimodified porcine donor hearts (lacking αGal epitopes and expressing human CD46 as well as human thrombomodulin) and appropriate immunosuppression, two steps were key to success: 1) nonischemic preservation of the donor hearts by perfusion with oxygenated hyperoncotic blood-based solution; and 2) prevention of detrimental xeno-heart overgrowth by early weaning of cortisone, lowering of blood pressure and treatment with the mTOR inhibiting prodrug temsirolimus (Längin et al., 2018).

Heart transplantation is the only cure for patients with terminal cardiac failure, but the supply of human donor organs does not meet the clinical need. Xenotransplantation of genetically modified pig hearts is a potential alternative as demonstrated by long-term survival (up to 945 d) of genetically multimodified pig hearts (GGTA1 KO, hCD46/hTBM transgenic) after heterotopic abdominal transplantation in baboons (Mohiuddin et al., 2016). Although this model demonstrated long-term acceptance of discordant cardiac xenotransplants with safe immunosuppression, their life supporting function remained to be proven. Therefore, Längin et al. (2018) used the same genetic background of donor pigs and adapted the immunosuppressive regimen developed by Mohiuddin et al. (2016) to perform a series of orthotopic heart transplantation (= heart replacement) experiments in baboons, finally resulting in consistent long-term success with survival times up to 195 d (Figure 3). The most essential improvements were 1) specific perfusion preservation of the xeno-hearts after explantation and during implantation with 8 °C-oxygenated hyperoncotic cardioplegic solution containing nutrition, hormones, and erythrocytes; and 2) post-transplantation growth control of the xeno-hearts by early weaning of glucocorticoids, lowering the recipients’ blood pressure, and inhibition of mTOR (mechanistic target of rapamycin) activation to counteract cardiomyocyte hypertrophy. Consistent life-supporting function of xeno-hearts for up to 195 d in the most relevant and stringent preclinical animal model is a milestone on the way to clinical cardiac xenotransplantation (Längin et al., 2018).

## Conclusions and Perspectives

Recent studies of life-supporting cardiac (Längin et al., 2018) and kidney xenotransplantation (survival > 400 d; Kim et al., 2019) in nonhuman primates have achieved survival times that the initiation of clinical xenotransplantation trials may be justified. This requires an internationally accepted regulatory framework covering safety and quality standards of donor pigs, requirements for preclinical data, selection and information of trial participants, post-transplant long-term patient follow-up, and storage of appropriate pre- and post-procedure specimens from donor pigs and patients. Pertinent recommendations from the Third WHO Global Consultation on Regulatory Requirements for Xenotransplantation Clinical Trials (Changsha, China, December 12–14, 2018) will be published as The 2018 Changsha Communiqué.

## About the Authors

**Eckhard Wolf** studied Veterinary Medicine at the LMU Munich, Germany. He is Head of the Institute for Molecular Animal Breeding and Biotechnology, Director of the Laboratory for Functional Genome Analysis, and Director of the Center for Innovative Medical Models at LMU Munich. His lab is specialized in the generation and characterization of genetically engineered pigs as models for human diseases (diabetes mellitus and rare monogenic diseases) and as organ donors for xenotransplantation. Wolf is Speaker of the DFG-funded Transregional Collaborative Research Center (TRR) 127 “Biology of xenogeneic cell, tissue and organ transplantation – from bench to bedside.”

**Elisabeth Kemter** studied Veterinary Medicine at the LMU Munich, Germany. She is a Senior Scientist at the Institute for Molecular Animal Breeding and Biotechnology and Center for Innovative Medical Models at LMU Munich with extensive experience in molecular and cell biology and Veterinary Specialization degrees in Laboratory Animal Science and in Veterinary Pathology. She is Principal Investigator in the DFG-funded TRR 127 “Biology of xenogeneic cell, tissue and organ transplantation – from bench to bedside,” where she is leading the porcine pancreatic islet program.

**Nikolai Klymiuk** studied Biochemistry at the University of Vienna, Austria. He has been a Senior Scientist at the Institute for Molecular Animal Breeding and Biotechnology and Center for Innovative Medical Models at LMU Munich since 2005 and focused his research on the design and generation of large animal models for biomedical research. Since May 2019, he is Associate Professor for Cardiovascular Translation in Large Animal Models at the Technical University Munich. He is Principal Investigator in the DFG-funded TRR 127 “Biology of xenogeneic cell, tissue and organ transplantation – from bench to bedside,” where he is leading the porcine genetic engineering program.

**Bruno Reichart** is a German cardiothoracic surgeon who performed Germany’s first successful combined heart-lung transplantation in 1983. In 1984, he succeeded Prof. Christiaan Barnard at Groote Schuur Hospital in Cape Town and was appointed President of the International Society for Heart and Lung Transplantation from 1988 to 1990. He returned to Munich in 1990 as Director of the Cardiac Surgery Clinic at the Klinikum Großhadern of LMU Munich, a position he held till 2011. He has been active in experimental research in discordant xenotransplantation from 1998, currently as Co-Speaker of the DFG-funded TRR 127 “Biology of xenogeneic cell, tissue and organ transplantation – from bench to bedside.”

## References

[CIT0001] Aron Badin R., Vadori M., Vanhove B., Nerriere-Daguin V., Naveilhan P., Neveu I., Jan C., Lévèque X., Venturi E., Mermillod P., et al. 2016 Cell therapy for parkinson’s disease: a translational approach to assess the role of local and systemic immunosuppression. Am. J. Transplant. 16:2016–2029. doi:10.1111/ajt.13704 26749114

[CIT0002] Bähr A., Käser T., Kemter E., Gerner W., Kurome M., Baars W., Herbach N., Witter K., Wünsch A., Talker S. C., et al. 2016 Ubiquitous LEA29Y expression blocks T cell co-stimulation but permits sexual reproduction in genetically modified pigs. Plos One. 11:e0155676. doi:10.1371/journal.pone.0155676 27175998 PMC4866763

[CIT0003] Bartlett S. T., Markmann J. F., Johnson P., Korsgren O., Hering B. J., Scharp D., Kay T. W., Bromberg J., Odorico J. S., Weir G. C., et al. 2016 Report from IPITA-TTS opinion leaders meeting on the future of β-cell replacement. Transplantation. 100(Suppl 2):S1–44. doi:10.1097/TP.0000000000001055 PMC4741413 26840096

[CIT0004] Bottino R., Wijkstrom M., van der Windt D. J., Hara H., Ezzelarab M., Murase N., Bertera S., He J., Phelps C., Ayares D., et al. 2014 Pig-to-monkey islet xenotransplantation using multi-transgenic pigs. Am. J. Transplant. 14:2275–2287. doi:10.1111/ajt.12868 25220221 PMC4169326

[CIT0005] Buermann A., Petkov S., Petersen B., Hein R., Lucas-Hahn A., Baars W., Brinkmann A., Niemann H., and Schwinzer R. 2018 Pigs expressing the human inhibitory ligand PD-L1 (CD 274) provide a new source of xenogeneic cells and tissues with low immunogenic properties. Xenotransplantation. 25:e12387. doi:10.1111/xen.12387 29446180

[CIT0006] Byrne G. W., McGregor C. G. A., and Breimer M. E.. 2015 Recent investigations into pig antigen and anti-pig antibody expression. Int. J. Surg. 23(Pt B):223–228. doi:10.1016/j.ijsu.2015.07.724 26306769 PMC4684721

[CIT0007] Cooper D. K., Ekser B., Ramsoondar J., Phelps C., and Ayares D. 2016 The role of genetically engineered pigs in xenotransplantation research. J. Pathol. 238:288–299. doi:10.1002/path.4635 26365762 PMC4689670

[CIT0008] Cowan P. J., and Robson S. C.. 2015 Progress towards overcoming coagulopathy and hemostatic dysfunction associated with xenotransplantation. Int. J. Surg. 23(Pt B):296–300. doi:10.1016/j.ijsu.2015.07.682 26220018

[CIT0009] Cozzi E., and White D. J.. 1995 The generation of transgenic pigs as potential organ donors for humans. Nat. Med. 1:964–966. 7585226 10.1038/nm0995-964

[CIT0010] Denner J 2018 Why was PERV not transmitted during preclinical and clinical xenotransplantation trials and after inoculation of animals? Retrovirology. 15:28. doi:10.1186/s12977-018-0411-8 29609635 PMC5879552

[CIT0011] Diamond L. E., Quinn C. M., Martin M. J., Lawson J., Platt J. L., and Logan J. S. 2001 A human CD46 transgenic pig model system for the study of discordant xenotransplantation. Transplantation. 71:132–142. 11211178 10.1097/00007890-200101150-00021

[CIT0012] Dieckhoff B., Petersen B., Kues W. A., Kurth R., Niemann H., and Denner J. 2008 Knockdown of porcine endogenous retrovirus (PERV) expression by PERV-specific shrna in transgenic pigs. Xenotransplantation. 15:36–45. doi:10.1111/j.1399-3089.2008.00442.x 18333912

[CIT0013] Egerer S., Fiebig U., Kessler B., Zakhartchenko V., Kurome M., Reichart B., Kupatt C., Klymiuk N., Wolf E., Denner J., et al. 2018 Early weaning completely eliminates porcine cytomegalovirus from a newly established pig donor facility for xenotransplantation. Xenotransplantation. 25:e12449. doi:10.1111/xen.12449 30264883

[CIT0014] Estrada J. L., Martens G., Li P., Adams A., Newell K. A., Ford M. L., Butler J. R., Sidner R., Tector M., and Tector J. 2015 Evaluation of human and non-human primate antibody binding to pig cells lacking GGTA1/CMAH/β4galnt2 genes. Xenotransplantation. 22:194–202. doi:10.1111/xen.12161 25728481 PMC4464961

[CIT0015] Fischer K., Kraner-Scheiber S., Petersen B., Rieblinger B., Buermann A., Flisikowska T., Flisikowski K., Christan S., Edlinger M., Baars W., et al. 2016 Efficient production of multi-modified pigs for xenotransplantation by ‘combineering’, gene stacking and gene editing. Sci. Rep. 6:29081. doi:10.1038/srep29081 27353424 PMC4926246

[CIT0016] Fishman J. A 2018 Infectious disease risks in xenotransplantation. Am. J. Transplant. 18:1857–1864. doi:10.1111/ajt.14725 29513380

[CIT0017] Fodor W. L. et al. 1994 Expression of a functional human complement inhibitor in a transgenic pig as a model for the prevention of xenogeneic hyperacute organ rejection. Proc. Natl. Acad. Sci. U S A. 91:11153–11157. 7526391 10.1073/pnas.91.23.11153 PMC45185

[CIT0018] Hara H., Witt W., Crossley T., Long C., Isse K., Fan L., Phelps C. J., Ayares D., Cooper D. K., Dai Y., et al. 2013 Human dominant-negative class II transactivator transgenic pigs - effect on the human anti-pig T-cell immune response and immune status. Immunology. 140:39–46. doi:10.1111/imm.12107 23566228 PMC3809704

[CIT0019] Hauschild J. et al. 2011 Efficient generation of a biallelic knockout in pigs using zinc-finger nucleases. Proc. Natl. Acad. Sci. U S A. 108:12013–12017. 21730124 10.1073/pnas.1106422108 PMC3141985

[CIT0020] Hawthorne W. J., Salvaris E. J., Phillips P., Hawkes J., Liuwantara D., Burns H., Barlow H., Stewart A. B., Peirce S. B., Hu M., et al. 2014 Control of IBMIR in neonatal porcine islet xenotransplantation in baboons. Am. J. Transplant. 14:1300–1309. doi:10.1111/ajt.12722 24842781 PMC4204157

[CIT0021] Iwase H., Ekser B., Hara H., Phelps C., Ayares D., Cooper D. K., and Ezzelarab M. B. 2014 Regulation of human platelet aggregation by genetically modified pig endothelial cells and thrombin inhibition. Xenotransplantation. 21:72–83. doi:10.1111/xen.12073 24188473 PMC4010578

[CIT0022] Kemter E., Cohrs C. M., Schäfer M., Schuster M., Steinmeyer K., Wolf-van Buerck L., Wolf A., Wuensch A., Kurome M., Kessler B., et al. 2017 INS-eGFP transgenic pigs: a novel reporter system for studying maturation, growth and vascularisation of neonatal islet-like cell clusters. Diabetologia. 60:1152–1156. doi:10.1007/s00125-017-4250-2 28315950

[CIT0023] Kemter E., Denner J., and Wolf E. 2018 Will genetic engineering carry xenotransplantation of pig islets to the clinic? Curr. Diab. Rep. 18:103. doi:10.1007/s11892-018-1074-5 30229378

[CIT0024] Kemter E., and Wolf E.. 2018 Recent progress in porcine islet isolation, culture and engraftment strategies for xenotransplantation. Curr. Opin. Organ Transplant. 23:633–641. doi:10.1097/MOT.0000000000000579 30247169

[CIT0025] Kim S. C., Mathews D. V., Breeden C. P., Higginbotham L. B., Ladowski J., Martens G., Stephenson A., Farris A. B., Strobert E. A., Jenkins J., et al. 2019 Long-term survival of pig-to-rhesus macaque renal xenografts is dependent on CD4 T cell depletion. Am. J. Transplant. 2019 Mar 1. doi:10.1111/ajt.15329. [Epub ahead of print]. 10.1111/ajt.15329 PMC6658347 30821922

[CIT0026] Klose R., Kemter E., Bedke T., Bittmann I., Kelsser B., Endres R., Pfeffer K., Schwinzer R., and Wolf E. 2005 Expression of biologically active human TRAIL in transgenic pigs. Transplantation. 80:222–230. 16041267 10.1097/01.tp.0000164817.59006.c2

[CIT0027] Klymiuk N., Aigner B., Brem G., and Wolf E. 2010 Genetic modification of pigs as organ donors for xenotransplantation. Mol. Reprod. Dev. 77:209–221. doi:10.1002/mrd.21127 19998476

[CIT0028] Klymiuk N., van Buerck L., Bähr A., Offers M., Kessler B., Wuensch A., Kurome M., Thormann M., Lochner K., Nagashima H., et al. 2012 Xenografted islet cell clusters from INSLEA29Y transgenic pigs rescue diabetes and prevent immune rejection in humanized mice. Diabetes. 61:1527–1532. doi:10.2337/db11-1325 22522620 PMC3357306

[CIT0029] Kwon D. J., Kim D. H., Hwang I. S., Kim D. E., Kim H. J., Kim J. S., Lee K., Im G. S., Lee J. W., and Hwang S. 2017 Generation of α-1,3-galactosyltransferase knocked-out transgenic cloned pigs with knocked-in five human genes. Transgenic Res. 26:153–163. doi:10.1007/s11248-016-9979-8 27554374 PMC5243873

[CIT0030] Kwon D. N., Lee K., Kang M. J., Choi Y. J., Park C., Whyte J. J., Brown A. N., Kim J. H., Samuel M., Mao J., et al. 2013 Production of biallelic CMP-Neu5Ac hydroxylase knock-out pigs. Sci. Rep. 3:1981. doi:10.1038/srep01981 23760311 PMC4070623

[CIT0031] Längin M., Mayr T., Reichart B., Michel S., Buchholz S., Guethoff S., Dashkevich A., Baehr A., Egerer S., Bauer A., et al. 2018 Consistent success in life-supporting porcine cardiac xenotransplantation. Nature. 564:430–433. doi:10.1038/s41586-018-0765-z 30518863

[CIT0032] Le Bas-Bernardet S. et al. 2011 Xenotransplantation of galactosyl-transferase knockout, CD55, CD59, CD39, and fucosyl-transferase transgenic pig kidneys into baboons. Transplant. Proc. 43:3426–3430. 22099813 10.1016/j.transproceed.2011.09.024

[CIT0033] Lee S. C., Lee H., Oh K. B., Hwang I. S., Yang H., Park M. R., Ock S. A., Woo J. S., Im G. S., and Hwang S. 2017 Production and breeding of transgenic cloned pigs expressing human CD73. Dev. Reprod. 21:157–165. doi:10.12717/DR.2017.21.2.157 28785737 PMC5532308

[CIT0034] Lin C. C., Ezzelarab M., Hara H., Long C., Lin C. W., Dorling A., and Cooper D. K. 2010 Atorvastatin or transgenic expression of TFPI inhibits coagulation initiated by anti-nongal igg binding to porcine aortic endothelial cells. J. Thromb. Haemost. 8:2001–2010. doi:10.1111/j.1538-7836.2010.03950.x 20553382 PMC2965779

[CIT0035] Lutz A. J., Li P., Estrada J. L., Sidner R. A., Chihara R. K., Downey S. M., Burlak C., Wang Z. Y., Reyes L. M., Ivary B., et al. 2013 Double knockout pigs deficient in N-glycolylneuraminic acid and galactose α-1,3-galactose reduce the humoral barrier to xenotransplantation. Xenotransplantation. 20:27–35. doi:10.1111/xen.12019 23384142

[CIT0036] Martin C., Plat M., Nerriére-Daguin V., Coulon F., Uzbekova S., Venturi E., Condé F., Hermel J. M., Hantraye P., Tesson L., et al. 2005 Transgenic expression of CTLA4-ig by fetal pig neurons for xenotransplantation. Transgenic Res. 14:373–384. 16201404 10.1007/s11248-004-7268-4

[CIT0037] Miyagawa S., Nakatsu S., Nakagawa T., Kondo A., Matsunami K., Hazama K., Yamada J., Tomonaga K., Miyazawa T., and Shirakura R. 2005 Prevention of PERV infections in pig to human xenotransplantation by the RNA interference silences gene. J. Biochem. 137:503–508. doi:10.1093/jb/mvi059. 15858174

[CIT0038] Mohiuddin M. M., Singh A. K., Corcoran P. C., Thomas M. L. 3rd, Clark T., Lewis B. G., Hoyt R. F., Eckhaus M., Pierson R. N. 3rd, Belli A. J., et al. 2016 Chimeric 2C10R4 anti-CD40 antibody therapy is critical for long-term survival of GTKO.hcd46.htbm pig-to-primate cardiac xenograft. Nat. Commun. 7:11138. doi:10.1038/ncomms11138 27045379 PMC4822024

[CIT0039] Mulder A., Kardol M. J., Arn J. S., Eijsink C., Franke M. E., Schreuder G. M., Haasnoot G. W., Doxiadis I. I., Sachs D. H., Smith D. M., et al. 2010 Human monoclonal HLA antibodies reveal interspecies crossreactive swine MHC class I epitopes relevant for xenotransplantation. Mol. Immunol. 47:809–815. doi:10.1016/j.molimm.2009.10.004 19931911

[CIT0040] Niu D., Wei H. J., Lin L., George H., Wang T., Lee I. H., Zhao H. Y., Wang Y., Kan Y., Shrock E., et al. 2017 Inactivation of porcine endogenous retrovirus in pigs using CRISPR-cas9. Science. 357:1303–1307. doi:10.1126/science.aan4187 28798043 PMC5813284

[CIT0041] Oropeza M., Petersen B., Carnwath J. W., Lucas-Hahn A., Lemme E., Hassel P., Herrmann D., Barg-Kues B., Holler S., Queisser A. L., et al. 2009 Transgenic expression of the human A20 gene in cloned pigs provides protection against apoptotic and inflammatory stimuli. Xenotransplantation. 16:522–534. doi:10.1111/j.1399-3089.2009.00556.x 20042052

[CIT0042] Petersen B., Ramackers W., Lucas-Hahn A., Lemme E., Hassel P., Queisser A. L., Herrmann D., Barg-Kues B., Carnwath J. W., Klose J., et al. 2011 Transgenic expression of human heme oxygenase-1 in pigs confers resistance against xenograft rejection during ex vivo perfusion of porcine kidneys. Xenotransplantation. 18:355–368. doi:10.1111/j.1399-3089.2011.00674.x 22168142

[CIT0043] Phelps C. J., Ball S. F., Vaught T. D., Vance A. M., Mendicino M., Monahan J. A., Walters A. H., Wells K. D., Dandro A. S., Ramsoondar J. J., et al. 2009 Production and characterization of transgenic pigs expressing porcine CTLA4-ig. Xenotransplantation. 16:477–485. doi:10.1111/j.1399-3089.2009.00533.x 20042047

[CIT0044] Phelps C. J., Koike C., Vaught T. D., Boone J., Wells K. D., Chen S. H., Ball S., Specht S. M., Polejaeva I. A., Monahan J. A., et al. 2003 Production of alpha 1,3-galactosyltransferase-deficient pigs. Science. 299:411–414. doi:10.1126/science.1078942 12493821 PMC3154759

[CIT0045] Ramsoondar J., Vaught T., Ball S., Mendicino M., Monahan J., Jobst P., Vance A., Duncan J., Wells K., and Ayares D. 2009 Production of transgenic pigs that express porcine endogenous retrovirus small interfering rnas. Xenotransplantation. 16:164–180. doi:10.1111/j.1399-3089.2009.00525.x 19566656

[CIT0046] Reyes L. M., Estrada J. L., Wang Z. Y., Blosser R. J., Smith R. F., Sidner R. A., Paris L. L., Blankenship R. L., Ray C. N., Miner A. C., et al. 2014 Creating class I MHC-null pigs using guide RNA and the cas9 endonuclease. J. Immunol. 193:5751–5757. doi:10.4049/jimmunol.1402059 25339675 PMC5922270

[CIT0047] Tena A., Kurtz J., Leonard D. A., Dobrinsky J. R., Terlouw S. L., Mtango N., Verstegen J., Germana S., Mallard C., Arn J. S., et al. 2014 Transgenic expression of human CD47 markedly increases engraftment in a murine model of pig-to-human hematopoietic cell transplantation. Am. J. Transplant. 14:2713–2722. doi:10.1111/ajt.12918 25278264 PMC4236244

[CIT0048] Vadori M., and Cozzi E.. 2015 The immunological barriers to xenotransplantation. Tissue Antigens 86:239–253. doi:10.1111/tan.12669 26381044

[CIT0049] Wang Y., Yang H. Q., Jiang W., Fan N. N., Zhao B. T., Ou-Yang Z., Liu Z. M., Zhao Y., Yang D. S., Zhou X. Y., et al. 2015 Transgenic expression of human cytoxic T-lymphocyte associated antigen4-immunoglobulin (hCTLA4Ig) by porcine skin for xenogeneic skin grafting. Transgenic Res. 24:199–211. doi:10.1007/s11248-014-9833-9 25236862

[CIT0050] Weiss E. H., Lilienfeld B. G., Müller S., Müller E., Herbach N., Kessler B., Wanke R., Schwinzer R., Seebach J. D., Wolf E., et al. 2009 HLA-E/human beta2-microglobulin transgenic pigs: protection against xenogeneic human anti-pig natural killer cell cytotoxicity. Transplantation. 87:35–43. doi:10.1097/TP.0b013e318191c784 19136889

[CIT0051] Wheeler D. G., Joseph M. E., Mahamud S. D., Aurand W. L., Mohler P. J., Pompili V. J., Dwyer K. M., Nottle M. B., Harrison S. J., d’Apice A. J., et al. 2012 Transgenic swine: expression of human CD39 protects against myocardial injury. J. Mol. Cell. Cardiol. 52:958–961. doi:10.1016/j.yjmcc.2012.01.002 22269791 PMC3327755

[CIT0052] Wolf-van Buerck L. et al. 2017 LEA29Y expression in transgenic neonatal porcine islet-like cluster promotes long-lasting xenograft survival in humanized mice without immunosuppressive therapy. Scientific Reports. 7:3572. 28620237 10.1038/s41598-017-03913-4 PMC5472587

[CIT0053] Wuensch A., Baehr A., Bongoni A. K., Kemter E., Blutke A., Baars W., Haertle S., Zakhartchenko V., Kurome M., Kessler B., et al. 2014 Regulatory sequences of the porcine THBD gene facilitate endothelial-specific expression of bioactive human thrombomodulin in single- and multitransgenic pigs. Transplantation. 97:138–147. doi:10.1097/TP.0b013e3182a95cbc 24150517

[CIT0054] Yan J. J., Yeom H. J., Jeong J. C., Lee J. G., Lee E. W., Cho B., Lee H. S., Kim S. J., Hwang J. I., Kim S. J., et al. 2016 Beneficial effects of the transgenic expression of human stnf-αr-fc and HO-1 on pig-to-mouse islet xenograft survival. Transpl. Immunol. 34:25–32. doi:10.1016/j.trim.2016.01.002 26777482

[CIT0055] Yang L., Güell M., Niu D., George H., Lesha E., Grishin D., Aach J., Shrock E., Xu W., Poci J., et al. 2015 Genome-wide inactivation of porcine endogenous retroviruses (pervs). Science. 350:1101–1104. doi:10.1126/science.aad1191 26456528

